# IFITMs from Mycobacteria Confer Resistance to Influenza Virus When Expressed in Human Cells

**DOI:** 10.3390/v7062759

**Published:** 2015-06-12

**Authors:** William J. Melvin, Temet M. McMichael, Nicholas M. Chesarino, Jocelyn C. Hach, Jacob S. Yount

**Affiliations:** Department of Microbial Infection and Immunity, Center for Microbial Interface Biology, the Ohio State University, Columbus, OH 43210, USA; E-Mails: wjamesmelvin@gmail.com (W.J.M.); temet.mcmichael@osumc.edu (T.M.M.); chesarino.1@osu.edu (N.M.C.); jocelyn.hach@gmail.com (J.C.H.)

**Keywords:** interferon induced transmembrane protein, IFITM3, influenza virus, virus restriction, palmitoylation

## Abstract

Interferon induced transmembrane proteins (IFITMs) found in vertebrates restrict infections by specific viruses. IFITM3 is known to be essential for restriction of influenza virus infections in both mice and humans. Vertebrate IFITMs are hypothesized to have derived from a horizontal gene transfer from bacteria to a primitive unicellular eukaryote. Since bacterial IFITMs share minimal amino acid identity with human IFITM3, we hypothesized that examination of bacterial IFITMs in human cells would provide insight into the essential characteristics necessary for antiviral activity of IFITMs. We examined IFITMs from *Mycobacterium avium* and *Mycobacterium abscessus* for potential antiviral activity. Both of these IFITMs conferred a moderate level of resistance to influenza virus in human cells, identifying them as functional homologues of IFITM3. Analysis of sequence elements shared by bacterial IFITMs and IFITM3 identified two hydrophobic domains, putative *S*-palmitoylation sites, and conserved phenylalanine residues associated with IFITM3 interactions, which are all necessary for IFITM3 antiviral activity. We observed that, like IFITM3, bacterial IFITMs were *S*-palmitoylated, albeit to a lesser degree. We also demonstrated the ability of a bacterial IFITM to co-immunoprecipitate with IFITM3 suggesting formation of a complex, and also visualized strong co-localization of bacterial IFITMs with IFITM3. However, the mycobacterial IFITMs lack the endocytic-targeting motif conserved in vertebrate IFITM3. As such, these bacterial proteins, when expressed alone, had diminished colocalization with cathepsin B-positive endolysosomal compartments that are the primary site of IFITM3-dependent influenza virus restriction. Though the precise evolutionary origin of vertebrate IFITMs is not known, our results support a model whereby transfer of a bacterial IFITM gene to eukaryotic cells may have provided a selective advantage against viral infection that was refined through the course of vertebrate evolution to include more robust signals for *S*-palmitoylation and localization to sites of endocytic virus trafficking.

## 1. Introduction

Interferon-induced transmembrane proteins (IFITMs) encoded by a variety of vertebrates, including pigs, chickens, bats, fish, mice, and humans, have been shown to restrict cellular infection by specific viruses [1,2,3,4,5,6]. IFITMs inhibit viruses that fuse within cellular endosomes [7,8] by preventing the formation of the viral fusion pore [9,10]. As a result of the block to viral entry through endosomes, virus is subsequently degraded in cathepsin-positive endolysosomes [6,11]. However, the molecular mechanisms by which IFITMs exert their antiviral functions and the precise evolutionary history of vertebrate IFITMs remain unknown [12,13].

The essentiality of mouse IFITM3 in restricting influenza virus infection was demonstrated by showing that IFITM3 knockout mice succumb to sublethal doses of virus and exhibit higher lung titers and more severe lung pathology than wild type mice [14,15]. Similarly, a nucleotide polymorphism in the human IFITM3 gene that is thought to affect proper IFITM3 RNA splicing has been associated with increased severity of influenza virus-associated disease [14,16,17,18]. Thus, IFITM3 is necessary for the innate immune restriction of influenza virus in mice and humans, and further understanding its biochemical properties may inspire new strategies for preventing or treating influenza and other IFITM-sensitive virus infections.

An extensive evolutionary study of IFITMs posited that vertebrate IFITMs originated from a horizontal gene transfer from bacteria to a common ancestor of choanoflagellates and metazoans [19]. Whether or not transfer of an IFITM gene provided an immediate antiviral selective advantage to eukaryotic cells is not known. Likewise, the function of IFITMs in bacteria has not been studied, though they have been annotated in numerous bacterial species including multiple mycobacterial strains [19]. Here, we examined the effect on influenza virus infection when mycobacterial IFITMs were expressed in human cells. Despite having roughly 20% amino acid identity with human or mouse IFITM3, we found that two divergent mycobacterial IFITMs each provided measurable inhibition of influenza virus infection of human cells. Surprisingly, we found that several essential characteristics necessary for the antiviral activity of IFITM3 are conserved in mycobacterial IFITMs, and speculate that the functional homology between mycobacterial IFITMs and vertebrate IFITM3 may be utilized to better understand both the evolutionary path of human IFITM3 and the amino acid determinants that make IFITM3 such a potent antiviral restriction factor.

## 2. Materials and Methods

### 2.1. Plasmids, Cloning, and Bioinformatics

Expression constructs in the pCMV-HA vector or pCMV-myc vectors (Clontech, Mountain View, CA, USA) encoding mouse and human HA-IFITM3, human myc-IFITM3, and mouse HA-IRGM1 have been previously described [4,20,21]. Untagged human IFITM3 was generated by removal of the HA tag from the pCMV-HA vector by site directed mutagenesis. mEmerald-Cathepsin B was a kind gift from Michael Davidson via Addgene (plasmid #54024). Human codon-optimized DNA sequences were generated from the amino acid sequences for MAV IFITM (UniProt A0QLS9) and MAB IFITM (UniProt B1MKV5). These sequences were purchased as synthetic Gene Strings from Life Technologies (Carlsbad, CA, USA). The complete sequences that were ordered including EcoRI and SalI restriction sites are shown below. These sequences were inserted into the pCMV-HA vector in frame with the HA tag via restriction sites. Phylogenetic tree analysis of the mycobacterial IFITMs, alignments, and calculations of percent amino acid identities were performed using Clustal Omega software (University College Dublin, Dublin, Ireland).

MAV IFITM
GAATTCCCATGACCCAACCACCTCCTCCCCCGCCACCACCAGGATATCCCCCACAACAGCCAGCCGCTCAGGCTCCTAATAACTATCTGGTGTGGTCTATCCTTGTCACTCTCTTCTGCTGCCTGCCGTTTGGCATTGTCGCTATCGTAAAGAGCTCTCAAGTGAACGGACTTTGGGCACAGGGTAGATATGCTGAGGCACAGGCCTCCGCAGACAGTGCCAAGAAATGGGTGATATGGAGCGCAGTTATAGGCGTCGTGGTGGGAATAATCTATGGAATCCTTATGGCCGTAGGCGCCCTCAACACAAATACAAACGCGGCCCTCGCCGCGATGTTTTAGTAAGTCGAC
        

MAB IFITM
GAATTCCCATGAGTGATGAAACCAAAAGCGACGAGCCTACAGGCGCTATCACCACACCGACCCCTCCTCCCCCACCGGCTCCTGCCTCTGTGACTGGCCCACCCAAACCCCCACCCACTAACGTGGGTTGGGCCGTCGCTAGCGTGATTTTTTTCTGGCCTCTGGCATTTAGCGCATTCACCAATGCACTGAATGTGACTCAGTTTTGGCTGACGGGGCAGTATGATCGGGCCCAGGAGTCTAGCGATCGGGCCAAGCTCCTGGGAAAGATTGCCCTCCTGACCGGGTTGGTACTGCTGTTCCTGTTCATCACCCTCCGCATTGCCTGCGCCATCTGGTGGCACTCACATGGTGGGGGATGGGGTCATCATGGCGGATGGCATAGGAGTTGGGACGACGGCGGGTGGGATGGCCCTGGCCCCATCGGGCCTATGGGTAGGCCGGGTCGCGACAACTAGTAAGTCGAC


### 2.2. Cells, Transfections, Palmitoylation Assay, and Virus Infections

HEK293T cells were obtained from the ATCC and were grown in DMEM supplemented with 10% FBS (Sigma, St. Louis, MO, USA) in a humidified incubator with 5% CO_2_ at 37 °C. For imaging experiments, cells were grown on sterilized glass coverslips in 12-well plates. For all other experiments, cells were grown in 12-well or 6-well plates. Cells were transfected overnight using LipoJet (SignaGen Laboratories, Gaithersburg, MD, USA) according to the manufacturer’s instructions. For biochemical experiments, 2 ug/well of each plasmid was transfected into cells grown in 6-well plates. For infection experiments, 1 ug/well of each plasmid was transfected into cells grown in 12-well plates, with the exception of plasmid encoding HA-MAB IFITM for which 0.5 ug/well was transfected. For examination of palmitoylation, transfected cells were labeled with 50 uM alk-16 for 1 h as previously described in detail [4,22,23]. Immunoprecipitiated proteins were then reacted with azido-rhodamine via click chemistry, subjected to SDS-PAGE, and visualized by fluorescent gel scanning [4,22,23]. Influenza viruses strains A/Puerto Rico/8/34 (H1N1, known as PR8) and the PR8 H3N2 reassortant virus strain known as X-31 were grown in 10-day embryonated chicken eggs for 48 h at 37 °C as described previously [24]. Sendai virus strain Cantell was grown in 10-day embryonated eggs for 40 h at 37 °C as described previously [25]. Cells were infected by replacing cellular media with 400 uL warm media containing virus. Infections were allowed to proceed for 6 h at 37 °C before collection of cells and analysis by flow cytometry.

### 2.3. Western Blotting, Immunoprecipitations, Immunofluorescence, and Flow Cytometry

For palmitoylation experiments, cells were lysed with 1% Brij Buffer (50 mM triethanolamine, 150 mM NaCl, 1% BrijO10 (Sigma), pH 7.4) containing EDTA-free protease inhibitor cocktail. For co-immunoprecipitation experiments, cells were lysed with 1% digitonin in PBS containing EDTA-free protease inhibitor cocktail. Immunoprecipitations were performed with either anti-HA or anti-myc EzView Affinity Gel (Sigma). For palmitoylation experiments, immunoprecipitations were washed 3 times with standard RIPA buffer (50 mM triethanolamine, 150 mM NaCl, 1% sodium deoxycholate, 1% Triton X100, 0.1% SDS, pH 7.4), and for co-immunoprecipitation experiments, the immunoprecipitations were washed 5 times with 0.1% digitonin in PBS. All detergents were purchased from Sigma. Western blotting was performed with anti-HA antibody (Covance, Dedham, MA, USA, HA.11, 1:1000) or anti-myc antibody (Developmental Studies Hybridoma Bank, Iowa City, IA, USA, 9E10, 1:1000). For immunofluorescence and flow cytometry, cells were fixed with 4% paraformaldehyde for 10 min, permeabilized with 0.1% Triton X100 in PBS for 10 min, and blocked with 2% FBS in PBS for 10 min. For immunofluorescence, cells were stained with the anti-HA or anti-myc antibodies described above that were directly labeled with Alexafluor 488 or 555 using 100 ug antibody kits from Life Technologies. Slides were mounted with Prolong Gold Antifade Reagent containing DAPI from Life Technologies and images were taken using an Olympus Fluoview FV10i confocal microscope. Quantification of Manders overlap coefficients was performed using the Just Another Colocalization Plugin (JACoP) for ImageJ software. For flow cytometry, cells were stained with anti-IFITM3 antibody (Cell Signaling, Boston, MA, USA) and anti-rabbit-Alexafluor-488 secondary antibody (Life Technologies), or with anti-HA antibody directly conjugated to Alexafluor-488 and anti-influenza NP antibody (Abcam, Cambridge, UK, ab20343) directly conjugated to Alexafluor-647 as previously described [20], or anti-Sendai virus antibody (MBL International Corporation, Woburn, MA, USA, PD029) with anti-rabbit-Alexafluor-647 secondary antibody (Life Technologies). Cells were analyzed using a BD Biosciences FACSCanto II flow cytometer (Franklin Lakes, NJ, USA) and Flowjo software (Ashland, OR, USA).

## 3. Results

### 3.1. Commonalities and Differences between IFITM3 and Mycobacterial IFITMs

**Figure 1:**
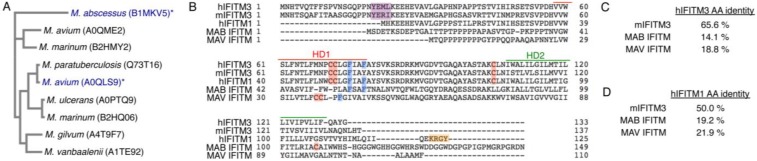
IFITMs from mycobacteria are divergent from vertebrate IFITMs, but possess certain shared amino acid sequence elements. (**A**) A phylogenetic tree of mycobacterial IFITMs generated with Clustal Omega alignments. The mycobacterial species and corresponding UniProt accession number for each IFITM is indicated. IFITMs from MAB (*M. abscessus*) and MAV (*M. avium*) that were chosen for further investigation are denoted with blue text and an asterisk; (**B**) Alignment of human (h) IFITM3, mouse (m) IFITM3, hIFITM1, MAB IFITM, and MAV IFITM. Hydrophobic domain (HD) 1 is highlighted with a red bar and HD2 is highlighted with a green bar. The YxxΦ motifs of hIFITM3 and mIFITM3 are shaded purple. Known *S*-palmitoylated cysteines in hIFITM3, mIFITM3, and hIFITM1, and potentially *S*-palmitoylated cysteines in MAB and MAV IFITMs are shaded red. Phenylalanines known to be involved in dimerization of IFITM3, and homologous phenylalanines in MAB and MAV IFITMs are shaded blue. The di-basic KRGY endocytic motif of hIFITM1 is shaded orange; (**C**) Amino acid percent identities as determined by Clustal alignment are shown comparing mycobacterial IFITMs with hIFITM3 or (**D**) hIFITM1. For (**C**) and (**D**), amino acid percent identities are also shown for mIFITM3 for comparison.

Evolutionary studies of the IFITM gene family have identified numerous IFITM genes in different vertebrates [19,26,27]. One study also identified more than 40 IFITM-related genes encoded by bacteria, and proposed that eukaryotic IFITMs derived from a horizontal gene transfer from bacteria to a single-celled ancestor of metazoans [19]. We sought to examine whether or not a bacterial IFITM could confer resistance to virus infection of human cells. Among the bacterial IFITM sequences previously identified, nine belong to mycobacterial strains [19]. A phylogenetic analysis of these nine amino acid sequences indicated that an IFITM from *Mycobacterium abscessus* (MAB) was significantly divergent from the majority of the remaining mycobacterial IFITMs (Figure 1A). For further analysis we chose the MAB IFITM and a *Mycobacterium avium* (MAV) IFITM as a representative of the more typical mycobacterial IFITMs (Figure 1A). ClustalO alignment of these two IFITMs along with human and mouse IFITM3 showed that the mycobacterial IFITMs share minimal sequence identity with human IFITM3 (Figure 1B,C, 14.1% identity for MAB IFITM and 18.8% for MAV IFITM). This is in contrast to the greater amino acid conservation shared between mouse and human IFITM3 (Figure 1B, 65.6%), which are known to be functional homologues. Since, like the mycobacterial IFITMs, human IFITM1 possesses a shortened N-terminus as compared to IFITM3, we also aligned the mycobacterial IFITMs with IFITM1 (Figure 1B). However, the amino acid conservation between IFITM1 and MAB IFITM was still only 19.2% and only 21.9% for MAV IFITM (Figure 1D). One important difference we noted between MAB and MAV IFITMs and IFITM3 is the lack of a YxxΦ motif in the N-terminus of the mycobacterial IFITMs (Figure 1B). We, and others, previously found that this tetrapeptide promotes the localization of IFITM3 to endosomes and lysosomes [20,28,29]. Likewise, the mycobacterial IFITMs also lack the C-terminal di-basic endocytic motif that is alternatively utilized by human IFITM1 [30] (Figure 1B).

Despite the major differences between the amino acid sequences of IFITM3 and mycobacterial IFITMs, a similar fundamental domain structure could be identified in each of these proteins. Two hydrophobic domains separated by approximately 30 amino acids are defining characteristics of IFITMs and were present in both MAV and MAB IFITMs (Figure 1B). Most vertebrate IFITMs also possess *S*-palmitoylated cysteines, including an *S*-palmitoylated di-cysteine motif, within the first hydrophobic domain [4,31,32] (Figure 1B). Interestingly, the di-cysteine motif is conserved within MAV IFITM, but not within MAB IFITM, though MAB IFITM possesses a cysteine in a location similar to a non-conserved *S*-palmitoylation site on murine IFITM1 [33] (Figure 1B). Likewise, IFITM3 has been reported to homo-dimerize and hetero-dimerize with IFITM2, and this interaction is promoted by the presence of two redundant phenylalanines within the first hydrophobic domain [34]. Interestingly, both MAB and MAV IFITMs have at least one phenylalanine within their first hydrophobic domains in positions similar to IFITM3 (Figure 1B). Thus, despite the low amino acid identity between mycobacterial IFITMs and IFITM3, their domain architecture is conserved along with several specific residues that are particularly critical for IFITM3 antiviral activity.

### 3.2. Mycobacterial IFITMs Provide Antiviral Activity When Expressed in Human Cells

**Figure 2:**
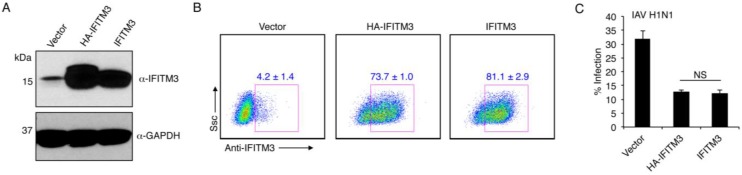
N-terminal HA-tagging of human IFITM3 does not affect its anti-influenza virus activity. (**A**) HEK293T cells were transfected with empty vector, or vector expressing human HA-IFITM3 or untagged human IFITM3. Cell lysates were examined by Western blotting with anti-IFITM3 antibodies and anti-GAPDH antibodies as a loading control; (**B**) Cells treated as in (**A**) were examined by flow cytometry staining with anti-IFITM3 antibodies. Cells are gated on IFITM3-positive cells and the blue numbers indicate the average of triplicate samples +/− standard deviation. Ssc, side scatter; (**C**) Cells treated as in (**A**) and (**B**) were infected with IAV H1N1 strain PR8 at an MOI of 1.0 or mock infected for 6 h. Cells were analyzed for percent infection by flow cytometry based on anti-IAV nucleoprotein staining in comparison to mock-infected controls. Average percent infection of triplicate samples in a representative experiment was graphed. Error bars indicate standard deviation. Results are representative of more than 5 similar experiments. NS, not significant by Student’s *t* test.

Given the important differences and striking similarities between the mycobacterial IFITMs and IFITM3 (Figure 1B), we sought to empirically determine if MAB and MAV IFITMs could confer antiviral activity when expressed in human cells. Since these IFITMs have never before been studied, antibodies for detection of their expression do not exist. Epitope tagging has been extensively used for the study of IFITMs [4,6,20,28,29,30,35,36,37,38,39,40,41], but it has been hypothesized that epitope tags may alter IFITM3 activity [7,8,13], though data demonstrating this has not been published. Thus, we first examined N-terminally HA-tagged and untagged human IFITM3 expressed from the same vector for their ability to inhibit influenza virus infection of HEK293T cells. This human cell line was utilized for our studies because it is highly infectable and capable of supporting the full life cycle of influenza virus [42], it expresses low levels of endogenous IFITM3 (Figure 2A,B), it is highly transfectable, and it has proved useful for distinguishing subtle differences in the activities of various IFITM mutants in several studies [4,9,20,28,29,33,36]. HEK293T cells robustly expressed both HA-IFITM3 and IFITM3 as examined by Western blotting with anti-IFITM3 antibodies (Figure 2A). Analysis at the single cell level by flow cytometry confirmed that only a small percentage of HEK293T cells express detectable levels of endogenous IFITM3, and that our transfection efficiencies for the IFITM3 constructs were greater than 70% (Figure 2B). Compared to the vector control transfection, both HA-IFITM3 and IFITM3 were able to significantly inhibit infection by influenza A virus (IAV) H1N1 strain PR8 as measured by anti-IAV nucleoprotein staining by flow cytometry [4,20,33,36]. No statistically significant difference was observed in the abilities of HA-IFITM3 and IFITM3 to inhibit infection (Figure 2C). Thus, we reasoned that utilization of HA-tagging of the mycobacterial IFITMs could provide an effective way to measure their expression without a likely effect on activity.

**Figure 3:**
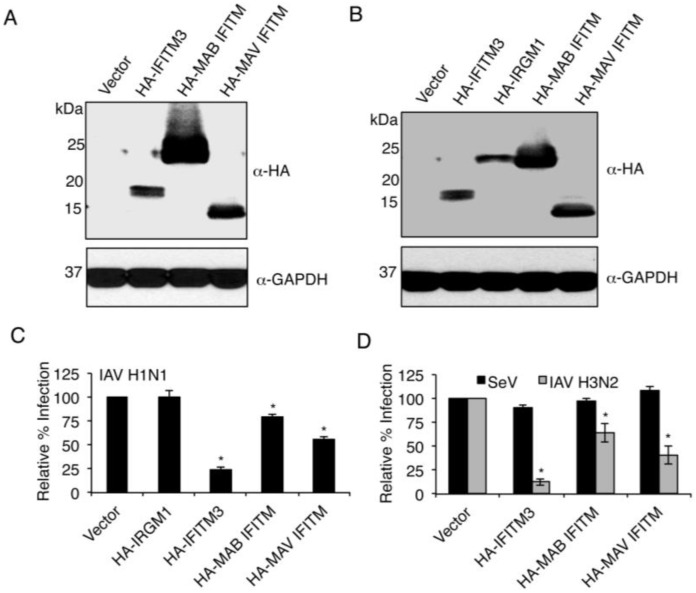
IFITMs from mycobacteria inhibit influenza virus infection when expressed in human cells. (**A**) HEK293T cells were transfected overnight with equal amounts empty vector or vector expressing human HA-IFITM3, HA-MAB IFITM3 or HA-MAV IFITM3. Lysates were analyzed by Western blotting with anti-HA antibody staining and anti-GAPDH staining as a loading control; (**B**) HEK293T cells were transfected overnight with equal amounts of empty vector, human HA-IFITM3, murine HA-IRGM1, or HA-MAV IFITM, or half the concentration of HA-MAB IFITM. Cell lysates were analyzed by Western blotting with anti-HA antibody staining and anti-GAPDH staining as a loading control; (**C**,**D**) Cells transfected as in **B** were infected with IAV H1N1 strain PR8 at an MOI of 2.5 or IAV H3N2 strain X31 or SeV at an MOI of 5.0. Cells were then collected and stained with anti-HA and anti-IAV nucleoprotein antibodies or anti-SeV antiserum. Cells were analyzed for percent infection by flow cytometry based on anti-IAV nucleoprotein or anti-SeV antibody staining in comparison to non-infected controls. The average percent infection for vector control cells was set to 1 for each infection. At least three independent experiments, each with triplicate measurements, were averaged and graphed. Error bars indicate standard errors of the means. * *p* < 0.01 compared to vector control by Student’s *t* test.

We synthesized codon optimized MAB and MAV IFITM genes for insertion into the pCMV-HA mammalian expression vector in-frame with the HA tag of the vector, and transfected these constructs into HEK293T cells. After transfection, we observed high expression of MAB IFITM, far greater than IFITM3 expression from the same vector, and a more similar expression level of MAV IFITM (Figure 3A). Thus, for infection experiments, we halved the amount of MAB IFITM plasmid utilized as compared to other plasmids, which provided a more comparable level of MAB IFITM (Figure 3B). These transfected cells were infected with IAV H1N1 for examination of antiviral activity. As negative controls we utilized empty pCMV-HA vector and overexpression of murine HA-IRGM1, which has been reported to be involved in antibacterial immunity [43,44], but has not been reported to inhibit influenza virus infection. We also utilized human HA-IFITM3 as a positive control. At 6 h post infection, we measured the percent of infected cells in each transfection condition by anti-IAV nucleoprotein staining analysis by flow cytometry [4,20,33,36]. As expected, HA-IRGM1 had no effect on infection as compared to the vector control, while HA-IFITM3 dramatically lowered the rate of infection (Figure 3C). Remarkably, both HA-MAB IFITM and HA-MAV IFITM also both significantly lowered the percentage of infected cells, with HA-MAV IFITM showing a more potent ability to restrict infection, though neither mycobacterial protein was as effective as HA-IFITM3 (Figure 3C). We also found that HA-MAB IFITM and HA-MAV IFITM were each able to inhibit infection by the IAV H3N2 strain X31 with similar efficacy to the inhibition of IAV H1N1 (Figure 3D). We then examined the specificity of this antiviral response by measuring the effect of these proteins on Sendai virus (SeV), which fuses at the cell surface and is not strongly inhibited by the endolysosomal perturbations induced by IFITM3 [33]. Importantly, the infection rate of SeV was not dramatically affected by expression of any of the IFITMs, confirming specificity of the antiviral effect of mycobacterial IFITMs, and that these mycobacterial proteins are not inherently toxic to cells (Figure 3D). Thus, the ability of the mycobacterial IFITMs to restrict IAV, along with the specificity of their virus restriction ability, identify MAB IFITM and MAV IFITM as functional homologues of IFITM3.

### 3.3. Mycobacterial IFITMs Are S-Palmitoylated in Human Cells

**Figure 4:**
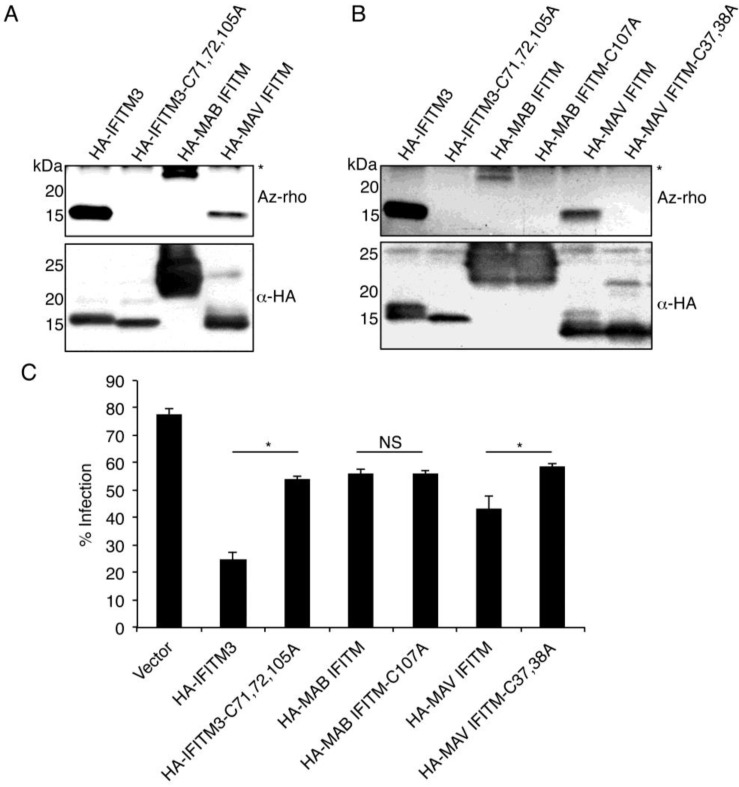
Mycobacterial IFITMs are modestly *S*-palmitoylated when expressed in human cells. (**A**–**C**) HEK293T cells were transfected overnight with the indicated murine HA-IFITM3 constructs or HA-tagged mycobacterial IFITM expression constructs or with empty vector control. (**A**,**B**) Cells were labeled for 1 h with 50 μM alk-16 or DMSO as a control and cell lysates were subjected to immunoprecipitation with anti-HA Affinity Gel prior to reaction with azido-rhodamine (az-rho) via click chemistry for fluorescence gel scanning in order to visualize levels of protein palmitoylation. Western blotting with anti-HA antibody was performed to allow comparison of protein loading. Data are representative of four experiments. * Denotes the antibody light chain used for immunoprecipitation that exhibits autofluorescence due to its high abundance; (**C**) Cells were infected for 6 h with IAV strain PR8 at an MOI of 2.5 and analyzed for percent infection by flow cytometry based on anti-IAV nucleoprotein staining in comparison to non-infected controls. The average percent infection of triplicate samples for a representative of three independent experiments was graphed. Error bars represent standard errors of the means. * *p* < 0.01 by Student’s *t* test comparing samples indicated by horizontal lines. NS, not significant by Student’s *t* test.

*S*-palmitoylation of IFITM3 is important for its proper membrane targeting and maximal antiviral activity [4,12,36]. Using a fluorescence-based chemical reporter assay [4,21,22,23,45,46], we examined whether or not MAB and MAV IFITMs were also palmitoylated. Both HA-MAB IFITM and HA-MAV IFITM showed fluorescent palmitoylation signal above that of the vector control and a non-palmitoylated mutant of HA-IFITM3, which both served as negative controls (Figure 4A). However, both of the mycobacterial IFITMs showed lower palmitoylation signal than HA-IFITM3, and, given the high level of immunoprecipitated HA-MAB IFITM, which is seen as a large and partially smeared band, its low palmitoylation signal suggests that it is only weakly palmitoylated (Figure 4A). HA-MAV IFITM, which showed a moderate level of palmitoylation as compared to HA-IFITM3 (Figure 4A), possesses a di-cysteine motif at amino acid positions 37 and 38 that is homologous to known *S*-palmitoylation sites on IFITM3 (Figure 1B). MAB IFITM possess only one cysteine at the C-terminal edge of its second hydrophobic domain (amino acid position 107, Figure 1B) that is reminiscent of a non-conserved *S*-palmitoylation site present on murine IFITM1 [33]. Using cysteine-to-alanine mutagenesis, we confirmed that MAV IFITM cysteines 37 and 38, and MAB IFITM cysteine 107 are indeed required for *S*-palmitoylation of these IFITMs (Figure 4B). We next tested the ability of these mutants to inhibit influenza virus infection. As previously reported, the palmitoylation-deficient mutant of IFITM3 lost a significant portion of its antiviral activity as compared to wild type IFITM3 [4,34,36] (Figure 4C). Similarly, non-palmitoylated MAV IFITM also partially lost antiviral activity, further suggesting a common mechanism of antiviral action between MAV IFITM and IFITM3 (Figure 4C). Consistent with the overall weak antiviral activity of MAB IFITM and its minimal *S*-palmitoylation (Figure 4A,B), mutation of cysteine 107 within this protein did not have a statistically significant effect on viral infection (Figure 4C). Overall these results are consistent with the assertion that strong *S*-palmitoylation of IFITMs is a driver of maximal antiviral activity [4,12,32].

### 3.4. MAV IFITM Co-Immunoprecipitates and Co-Localizes with IFITM3

**Figure 5:**
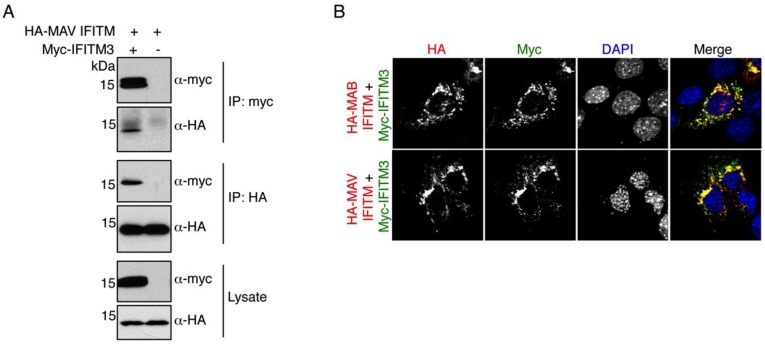
MAV IFITM co-immunoprecipitates and co-localizes with IFITM3. (**A**,**B**) HEK293T cells were transfected with the indicated constructs expressing HA-tagged mycobacterial IFITMs and human myc-IFITM3. (**A**) Cells were lysed and portions of the cell lysates were subjected to immunoprecipitation with either anti-HA or anti-myc Affinity Gel prior to SDS-PAGE and Western blot analysis with anti-HA or anti-myc antibodies. Western blotting of the cell lysates confirmed the equal input amounts of HA-MAV IFITM and the presence of myc-IFITM3; (**B**) Confocal microscopy imaging of mycobacterial IFITMs with myc-IFITM3 demonstrated their co-localization in cells. Anti-HA staining provided visualization of the mycobacterial IFITMs while anti-myc staining allowed visualization of IFITM3. DAPI staining was used to visualize nuclei. Merged images are shown with yellow regions indicating overlap between mycobacterial IFITMs and IFITM3.

IFITM3 was reported to homodimerize and also to heterodimerize with other IFITMs [34,47]. This dimerization was found to be dependent upon the presence of two phenylalanines within IFITM3’s first hydrophobic domain [34]. Mutation of either of these residues individually had little effect on dimerization or antiviral activity, but mutation of both residues eliminated dimerization and decreased antiviral activity [34]. These results suggested that these two phenylalanines serve a redundant purpose and that IFITM dimerization promotes antiviral activity. Interestingly, both MAB and MAV IFITMs possess at least one of these phenylalanines conserved within their first hydrophobic domains (Figure 1B). MAV-IFITM possesses one of these phenylalanines while MAB-IFITM possesses both. To provide evidence as to whether MAV IFITM might interact with IFITM3, we co-transfected HA-MAV IFITM with human myc-IFITM3 and examined their ability to co-immunoprecipate with one another. Indeed, we observed that MAV IFITM and IFITM3 co-precipitate (Figure 5A). Providing support for this finding that HA-MAV IFITM and IFITM3 may interact, HA-MAB IFITM and HA-MAV IFITM both strongly co-localize with Myc-IFITM3 in cells as visualized by confocal immunofluorescence microscopy (Figure 5B). Thus, the ability to interact may be a conserved aspect contributing to the functional homology between mycobacterial IFITMs and IFITM3.

### 3.5. Mycobacterial IFITMs Partially Localize to Cathepsin B-Positive Cellular Compartments

**Figure 6:**
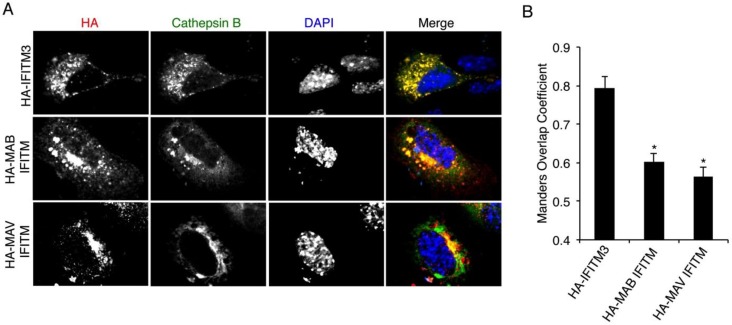
Mycobacterial IFITMs localize to lysosomes less efficiently than IFITM3. (**A**,**B**) HEK293T cells grown on glass coverslips were transfected with expression plasmids encoding human HA-IFITM3, HA-MAB IFITM, or HA-MAV IFITM along with plasmid expressing mEmerald-Cathepsin B. (**A**) Confocal microscopy imaging of anti-HA staining, which provided visualization of the IFITMs, and mEmerald imaging, which provided visualization of the cathepsin-positive lysosomal compartment. DAPI staining was used to visualize nuclei. Merged images are shown with yellow regions indicating overlap between IFITMs and Cathepsin B; (**B**) The Manders overlap coefficient for each indicated IFITM with Cathepsin B was quantified for at least 20 individual cells as visualized in (**A**) from two independent experiments. The average value was graphed and error bars represent standard errors of the means. * *p* < 0.0001 by Students *t* test compared to HA-IFITM3.

IFITM3 has been reported to co-localize with early and late endosomal markers and with markers of lysosomes and multi-vesicular bodies [6,9,10,11,28,29,36,48]. This localization is consistent with the ability of IFITM3 to prevent fusion of endocytosed influenza virus, and the subsequent degradation of the virus by lysosomal proteases [6,11]. The strong localization of IFITM3 to endolysosomes is due in large measure to the presence of a YxxΦ motif within its *N*-terminus that interacts with endocytic adaptor complexes, resulting in the effective trafficking of IFITM3 from the plasma membrane to endosomes [20,29]. This motif is highly conserved in vertebrate IFITMs [29], but is not present in the mycobacterial IFITMs, nor do they possess a sequence similar to the di-basic motif that directs the trafficking of human IFITM1 [30] (Figure 1B). We examined the co-localization of IFITM3 and mycobacterial IFITMs with mEmerald-tagged cathepsin B, a lysosomal protease. As expected, IFITM3 showed extensive co-localization with cathepsin B (Figure 6A). MAB and MAV IFITMs also both showed strong co-localization with cathepsin B, but these IFITMs also localized to cathepsin B-negative compartments (Figure 6A). Quantification of the overlap of each of the IFITMs with cathepsin B confirmed a significant decrease in the co-localization of mycobacterial IFITMs with cathepsin B-positive compartments as compared to IFITM3 (Figure 6B). Our results may indicate that the strong co-localization seen between mycobacterial IFITMs and IFITM3 in Figure 5B is indeed due to an interaction between these proteins since mycobacterial IFITMs and IFITM3 do not localize to precisely the same compartments when expressed in isolation (Figure 6). These results are consistent with the lack of an IFITM1- or IFITM3-homologous endocytic targeting motif within the mycobacterial IFITM amino acid sequences (Figure 1B) and, along with the weak *S*-palmitoylation that we observed for mycobacterial IFITMs compared to IFITM3 (Figure 4A,B), this localization phenotype may contribute to the reduced antiviral activity of the mycobacterial IFITMs (Figure 3C,D and Figure 4C).

## 4. Discussion

Though the exact evolutionary origin of vertebrate IFITMs is unknown, an IFITM with a splicing pattern that is conserved in all metazoans was identified in *Monosiga brevicollis* [19], a unicellular eukaryote considered to be the last unicellular ancestor of metazoans. This may suggest that IFITMs emerged in a common ancestor of metazoans and *Monosiga brevicollis*. IFITMs were also identified in multiple ancient bacteria species, and given the identifiable sequence similarities between bacterial IFITMs and metazoan IFITMs (Figure 1B), it was hypothesized that a horizontal gene transfer from bacteria introduced an IFITM gene into the single-celled metazoan ancestor discussed above [19]. Here, we chose two divergent bacterial IFITMs for introduction into human HEK293T cells. Both MAB and MAV IFITMs conferred enhanced resistance to influenza virus infection as compared to control transfections (Figure 3C,D). Confirming that the activity of these proteins is functionally homologous to IFITM3, these proteins, similarly to IFITM3, were unable to inhibit SeV infection (Figure 3D), and their level of activity correlated with their level of *S*-palmitoylation (Figure 4), which is a known positive regulatory modification of IFITM3 [4,12]. The functional homology of the bacterial IFITMs with IFITM3 could indicate that transfer of a bacterial IFITM to eukaryotes offered an advantage against virus infection that was refined through co-evolution with viruses. This hypothesis is in line with emerging data that IFITMs are antiviral in all animal species that have been tested and reported on to date [1,2,3,4,5,6]. This information also now provides a platform for examination of critical amino acid determinants of IFITM3 biology as discussed below.

Many aspects of IFITM3 cell biology that are not fully understood may possibly be clarified by study of the mycobacterial IFITMs. For example, IFITM3 is known to be optimally active when expressed at high levels in cells, and its turnover is promoted by post-translational ubiquitination [36]. However, we do not have a clear knowledge of the aspects of IFITM3 that control its cellular levels and ubiquitination. MAB IFITM was expressed at higher levels than MAV IFITM or IFITM3 when using the same amount of plasmid for transfections (Figure 3A). Thus, it will be interesting to determine what features of MAB IFITM allow its robust accumulation and whether or not it avoids ubiquitination and degradation. Such information may inspire strategies for increasing IFITM3 levels for the purpose of improving virus resistance. Likewise, despite possessing a di-cysteine motif in its first hydrophobic domain, MAV IFITM was less robustly palmitoylated than IFITM3 (Figure 4A,B). Interestingly, the MAV IFITM di-cysteine motif is shifted three positions deeper within the first hydrophobic domain compared to its location within IFITM3, and this is also true for most of the other mycobacterial IFITMs that possess the di-cysteine motif. This may suggest that the positioning of the di-cysteine motif within the hydrophobic domain is a critical determinant of its *S*-palmitoylation level and that positioning of this domain evolved toward optimal modification in vertebrates. Alternatively, other determinants may affect interaction with the as yet unidentified palmitoyltransferase enzyme that modifies IFITMs. Further interrogation of MAV IFITM may shed light on these important questions.

The precise mechanism of antiviral action of IFITMs is also currently unknown. Two potentially complementary models have been proposed for the anti-influenza virus activity of IFITM3. One, IFITM3 may directly alter fluidity of endolysosomal membranes [9,10,49] through the insertion of its intramembrane domain (the first hydrophobic domain) [12], and two, IFITM3 may alter membrane cholesterol levels by interacting with VAPA, a protein involved in cholesterol homeostasis [37]. Determining whether the mycobacterial IFITMs adopt an intramembrane topology or interact with VAPA may aid in distinguishing the importance of these two proposed models for IFITM3 antiviral activity. Beyond inhibition of viral entry, vertebrate IFITMs have also been suggested to inhibit HIV-1 by limiting virus replication [38,39], and by incorporating into newly produced virions leading to decreased infectivity [40,41]. Conversely, particular IFITMs have been reported to facilitate the cellular entry of a specific coronavirus [50]. It will be interesting to determine whether these additional functions of vertebrate IFITMs are shared by the mycobacterial IFITMs. Overall, we have identified two bacterial IFITMs as functional homologues of IFITM3, noted important similarities and differences between these proteins and IFITM3, and propose that further study of these proteins will provide greater understanding of IFITM3 post-translational regulation and antiviral activity.
